# Modeling and Optimal Design for a High Stability 2D Optoelectronic Angle Sensor

**DOI:** 10.3390/s19204409

**Published:** 2019-10-11

**Authors:** Zhenying Cheng, Liying Liu, Peng Xu, Ruijun Li, Kuang-Chao Fan, Hongli Li, Yongqing Wei

**Affiliations:** 1 School of Instrument Science and Opto-electronics Engineering, Hefei University of Technology, Hefei 230009, China; chengzhenying01@hfut.edu.cn (Z.C.); 2017110004@mail.hfut.edu.cn (L.L.); pengxu@mail.hfut.edu.cn (P.X.); fan@ntu.edu.tw (K.-C.F.); hlli@hfut.edu.cn (H.L.); yqwei_hfut@hfut.edu.cn (Y.W.); 2 School of Mechanical Engineering, Dalian University of Technology, Dalian 116024, China

**Keywords:** angle sensor, error model, drift, optimal design, stability

## Abstract

The structural deformations caused by environmental changes in temperature, vibration, and other factors are harmful to the stability of high precision measurement equipment. The stability and optimal design method of a 2D optoelectronic angle sensor have been investigated in this study. The drift caused by structural deformations of the angle sensor has been studied and a drift error model has been achieved. Key components sensitive to thermal and vibrational effects were identified by error sensitivity analysis and simulation. The mounts of key components were analyzed using finite element analysis software and optimized based on the concept of symmetric structures. Stability experiments for the original and optimized angle sensors have been carried out for contrast. As a result, the stability of the optimized angle sensor has been improved by more than 63%. It is verified that the modeling and optimal design method is effective and low-cost, which can also be applied to improve the stability of other sensors with much more complex principles and structures.

## 1. Introduction

In the past two decades, precision angle measurement technology has been found to be essential in various precision instruments, such as micro-coordinate measurement machines (CMMs), microprobes, nanopositioning stages, multi-degree-of-freedom measurement (MDFM) systems, and atomic force microscopes [1,2,3,4,5,6,7,8,9]. In general, precision angle measurement systems are widely used to detect micro angles or angular errors caused by tilting motions of moving objects. However, the measurement accuracy of most angle measurement systems is not high enough because of unstable working environments. Temperature fluctuation and vibration are the main environmental factors causing structural deformations of an angle sensor. Structural deformation is the most important reason for drift [10]. It has been proved that a small temperature change of 0.1 °C will cause 30 nm drift for a micro or nano system [11,12]. Therefore, structural deformations and drift errors caused by temperature fluctuation and vibration should be reduced to guarantee the stability of an angle measurement system.

Much work has been done on the stability of angle sensors. One of the most commonly used methods is to control the temperature by using a high precision chamber [4,13]. Although the stability of angle sensors can be improved effectively, doing so also makes the sensor too bulky and increases the cost. The other common method is using a laser coupled with a single-mode fiber to reduce the drift caused by the laser source [14,15,16]. However, other optical components and their mounts will also bring drifts. Some other methods that adopt complicated structures have also been developed to improve the angle sensor’s stability through compensation. Shimizu et al. developed an ultrasensitive angle sensor based on laser autocollimation, and adopted two single-cell photodiodes (SPDs) as the optical position detectors instead of a quadrant photodetector (QPD) [17]. Zhu et al. developed an angle sensor based on the laser autocollimation principle, for which the drift can be detected by another common-path angle sensor and corrected [6]. Huang et al. designed an angle sensor with a 2D hybrid mirror angle steering mount actuated by two piezoelectric actuators (PZTs), which can compensate for the drift of the angle sensor in real time [7]. A design concept that follows the symmetry structure principle was proposed by our group to improve the thermal stability of an angle sensor [18]. More quantitative details and comparisons of previous works are shown in Table 1.

From Table 1, it can be seen that the symmetrical design method is actually a potential way to improve the stability of simple angle sensors. However, if this method is used to improve the stability of complicated sensors with many components, each component or mount should be optimized and refabricated, after which the cost and time will greatly increase. Therefore, further investigation was still required to make this method more effective, more economic, and more universal. For example, we still needed to: (1) find the components that are more fragile and more sensitive than others to temperature fluctuation and vibration, along with their mounts; (2) find the relationship between the deformation of an optical component mount and the sensor’s drift; (3) find an effective way to reduce the drift caused by vibration.

In this study, a 2D optoelectronic angle sensor was taken as an example. Firstly, the error model calculating the angle between the sensor’s drift and the deformations of each component mount was built. Then, the components or mounts that are sensitive to temperature fluctuation and vibration were determined. Accordingly, the mounts of sensitive components were optimized and analyzed by finite element analysis software. Moreover, the correctness and effectiveness of the model were verified by comparative experiments.

## 2. Principle and Error Modeling of a 2D Angle Sensor

### 2.1. Principle

**Figure 1:**
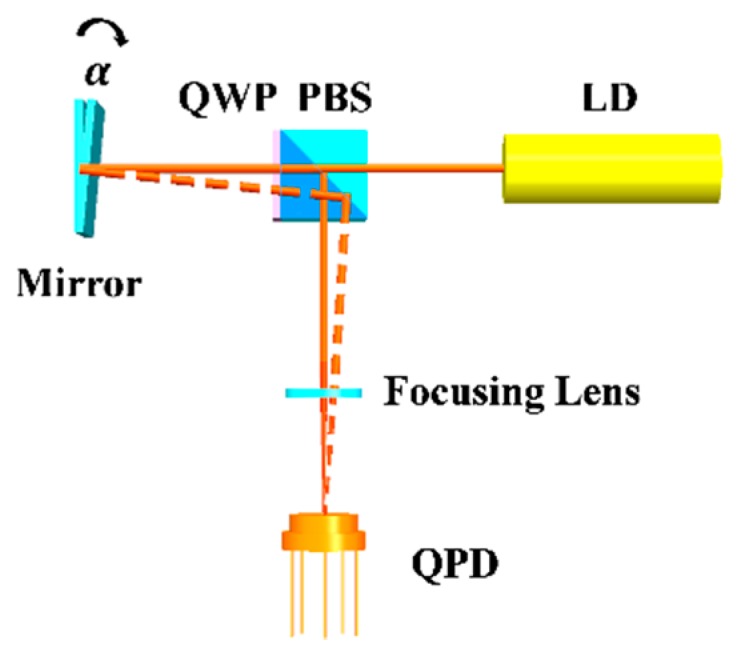
Optical configuration of the 2D angle sensor. Abbreviations: QWP = quarter-wave plate; PBS = polarizing beam splitter; LD = laser diode; QPD = quadrant photodetector.

The high-precision 2D angle sensor analyzed in this study was based on the laser autocollimation principle. The tilt angles of a plane mirror mounted on a measured object were detected as the displacement of a focused light spot on a photoelectric conversion device. The optical configuration of the 2D angle sensor is shown in Figure 1. To meet the needs of the sensor with high sensitivity and fast response, a quadrant photodetector (QPD; SPOT-4D, Centenary Materials Co., Ltd., Shanghai, China) was used as a light position sensing device. The polarized light emitted from the laser diode (LD; DI635-5-5, Huanic Co., Ltd., Shaanxi, China) is divided into P-polarized and S-polarized lights by a polarizing beam splitter (PBS). The P-polarized light is converted into S-polarized light by passing through the quarter-wave plate (QWP) twice, which is connected with the PBS. Then, the light was projected on the surface of the QPD by a focusing lens (FL), which came from a digital versatile disc (DVD) pick-up head (HOP-1000). An adjusting mechanism on which the plane mirror was mounted was used to tune the lights and focus them to the center of the QPD. When a small angle occurs on the plane mirror, the light spot on the surface of the QPD moves, the displacement of which can be detected by the QPD. The relationship between displacement *x* and measured angle *α* is described below.

(1) x=f⋅tan(2α)

where *f* is the focal length of the focusing lens.

### 2.2. Error Modeling

**Figure 2:**
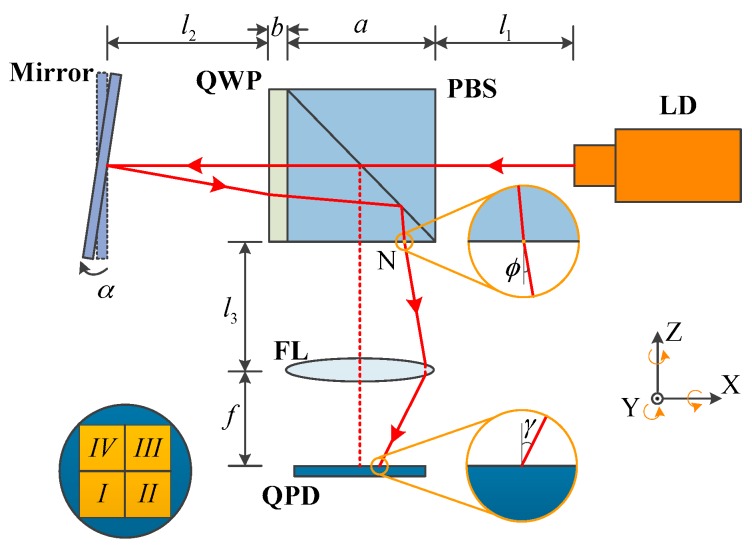
Diagram and parameters of the angle sensor.

Aiming to identify the components that are major contributors to the sensor’s stability, a coordinate system was established and the center point of the QPD was regarded as the coordinate origin. The coordinate of the center point of the focused light spot on the QPD can be expressed as (*x*, *y*). Assuming that the light from the LD initially passes through the centers of all optical components in sequence, an input angle *α* (*α_Y_*, *α_Z_*) was thereafter added on the plane mirror. The distances between each of the adjacent components, as well as the positive directions of rotation about three axes, are shown in Figure 2. Each of the components has an angle or displacement when the temperature changed or a vibration occurred. Therefore, the coordinate values (*x*, *y*) of the light spot on the QPD change.

Point N in Figure 2 locates the center position of the reflected light beam in the PBS. Here, *φ* (*φ_X_*, *φ_Y_*) is the angle between the light passing through PBS and the Z axis, and *γ* (*γ_X_*, *γ_Y_*) is the angle between the focused light and the Z axis. When LD or PBS has an angle or displacement, it can be obtained as:

(2) $$\varphi_{X}=-2\alpha_{Z}+\varepsilon_{LD-Z}+\varepsilon_{PBS-X}+\varepsilon_{PBS-Z},$$

(3) $$\varphi_{Y}=-2\alpha_{Y}+\varepsilon_{LD-Y}+2\varepsilon_{PBS-Y},$$

where *ε_LD_* (*ε_LD-X_*, *ε_LD-Y_*, *ε_LD-Z_*) and *ε_PBS_* (*ε_PBS-X_*, *ε_PBS-Y_*, *ε_PBS-Z_*) are angular errors of the LD and PBS, respectively; *δ_LD_* (*δ_LD-X_*, *δ_LD-Y_*, *δ_LD-Z_*) and *δ_PBS_* (*δ_PBS-X_*, *δ_PBS-Y_*, *δ_PBS-Z_*) are the displacement of LD and PBS, respectively. Therefore, the 3D coordinate values (*x_N_*, *y_N_*, *z_N_*) of point N can be expressed as follows in accordance with the law of refraction:

(4) $$x_{N}\approx [(a+b)\frac{n_{0}}{n_{1}}+\kappa ]\cdot [-\tan(2\alpha_{Y})+\tan\varepsilon_{LD-Y}]+\iota \tan\varepsilon_{LD-Y}+\frac{a}{2}\tan\varepsilon_{PBS-Y}-\delta_{LD-Z},$$

(5) $$y_{N}\approx [(a+b)\frac{n_{0}}{n_{1}}+\kappa ]\cdot [-\tan(2\alpha_{Z})+\tan\varepsilon_{LD-Z}]+\iota \tan\varepsilon_{LD-Z}+\frac{a}{2}\tan\varepsilon_{PBS-Z}+\delta_{LD-Y},$$

(6) $$z_{N}=f+l_{3}+x_{N}\cdot \tan\varepsilon_{PBS-Y}=f+l_{3}+y_{N}\cdot \tan\varepsilon_{PBS-X},$$

where $\iota =\sqrt{{\left(l_{1}+\delta_{LD-X}-\delta_{PBS-X}\right)}^{2}+\delta_{PBS-Z}^{2}}$, $\kappa =\sqrt{{\left(l_{2}+\delta_{PBS-X}\right)}^{2}+\delta_{PBS-Z}^{2}}$, *n*_0_ (=1), and *n*_1_ (=1.5) are the refractive indices of air and glass, respectively.

When an angular error *ε_FL_* (*ε_FL-X_*, *ε_FL-Y_*, *ε_FL-Z_*) and a displacement error *δ_FL_* (*δ_FL-X_*, *δ_FL-Y_*, *δ_FL-Z_*) occur on the FL at the same time, the included angle between the incident parallel light and the optical axis will change, and then the coordinates (*x*_1_, *y*_1_) of the light spot change in accordance with geometrical optics:

(7) $$x_{1}\approx 2f\cdot \tan\frac{\varepsilon_{FL-Y}}{2}+f\cdot \tan(\varphi_{Y}-\varepsilon_{FL-Y})+\delta_{FL-X}+\delta_{FL-Z}\cdot \tan(\gamma_{Y}+\varepsilon_{FL-Y}),$$

(8) $$y_{1}\approx 2f\cdot \tan\frac{\varepsilon_{FL-X}}{2}+f\cdot \tan(\varphi_{X}-\varepsilon_{FL-X})+\delta_{FL-Y}+\delta_{FL-Z}\cdot \tan(\gamma_{X}+\varepsilon_{FL-X}),$$

According to the autocollimation principle, the following equations can be obtained after considering the displacement errors for LD, PBS, and QPD, respectively:

(9) $$\tan\gamma_{Y}=\frac{(l_{3}-f+\delta_{PBS-Z}-2\delta_{FL-Z}+\delta_{QPD-Z})\cdot \tan\varphi_{Y}+x_{N}}{f},$$

(10) $$\tan\gamma_{X}=\frac{(l_{3}-f+\delta_{PBS-Z}-2\delta_{FL-Z}+\delta_{QPD-Z})\cdot \tan\varphi_{X}+y_{N}}{f},$$

Finally, we can obtain the coordinate values (*x*, *y*) of the light spot when an angular error *ε_QPD_* (*ε_QPD-X_*, *ε_QPD-Y_*, *ε_QPD-Z_*) and a displacement error *δ_QPD_* (*δ_QPD-X_*, *δ_QPD-Y_*, *δ_QPD-Z_*) occur on the QPD:

(11) $$x=\frac{x_{1}}{\cos\varepsilon_{QPD-Y}+\sin\varepsilon_{QPD-Y}\cdot \tan(\gamma_{Y}+\varepsilon_{FL-Y})}-\delta_{QPD-X}-\delta_{QPD-Z}\cdot \tan(\gamma_{Y}+\varepsilon_{FL-Y}),$$

(12) $$y=\frac{y_{1}}{\cos\varepsilon_{QPD-X}+\sin\varepsilon_{QPD-X}\cdot \tan(\gamma_{X}+\varepsilon_{FL-X})}-\delta_{QPD-Y}-\delta_{QPD-Z}\cdot \tan(\gamma_{X}+\varepsilon_{FL-X}),$$

The previous analysis shows that if additional angles and displacement are generated on the optical components, the center coordinates (*x*, *y*) of the light spot will be changed. As the plane mirror is usually mounted on the measured object, the influence of the plane mirror on stability is not considered here.

In the actual measurement process, the errors usually occur at the same time instead of separately. Thus, how (*x*, *y*) changes with so many error factors should be considered. Equations (13) and (14) can reflect the comprehensive results for the center coordinates of the light spot. Based on analysis and experimental results, the angular errors and the displacement errors of the optical components caused by temperature fluctuation and vibration are usually less than ± 60 arcsec and ± 3 μm, respectively. Therefore, the center coordinates (*x*, *y*) of the light spot can be simplified by omitting small values:

(13) $$x\approx -f\cdot \tan(2\alpha_{Y}-\varepsilon_{LD-Y}-2\varepsilon_{PBS-Y}-\varepsilon_{FL-Y})+\delta_{FL-X}-\delta_{QPD-X},$$

(14) $$y\approx -f\cdot \tan(2\alpha_{Z}-\varepsilon_{LD-Z}-\varepsilon_{PBS-X}-\varepsilon_{PBS-Z}-\varepsilon_{FL-X})+\delta_{FL-Y}-\delta_{QPD-Y},$$

## 3. Sensitivity Analysis and Optimization

### 3.1. Measurement Model and Error Sensitivity Analysis

Once the light spot is focused on the active areas on the QPD, four weak currents are generated. Two voltage signals that are proportional to the respective 2D angles can be obtained by applying appropriate resistances for each PD:

(15) $$U_{x}=\eta k\cdot [(P_{I}+P_{II})-(P_{III}+P_{IV})],$$

(16) $$U_{Y}=\eta k\cdot [(P_{I}+P_{IV})-(P_{II}+P_{III})],$$

where *P_i_* (*i* = *I*, *II*, *III* and *IV*) is the optical power converted by the *i*th PD, as shown in Figure 2; *η* is the photodiode conversion factor of this QPD, which is 0.42 A/W when the laser wavelength is 635 nm; and *k* is the amplification coefficient of the circuit, which is equal to 10^5^ in this sensor.

Supposing that the intensity of the light spot focused on the surface of the QPD obeys a uniform distribution, omitting the gap’s influence on the QPD, the relationship between the output voltages (*U_X_*, *U_Y_*) and the center coordinate values (*x*, *y*) of the light spot can be obtained [19]:

(17) $$U_{x}=4\eta kp\cdot (\frac{1}{2}x\cdot \sqrt{r^{2}-x^{2}}+\frac{r^{2}}{2}\arcsin\frac{x}{r}),$$

(18) $$U_{x}=4\eta kp\cdot (\frac{1}{2}y\cdot \sqrt{r^{2}-y^{2}}+\frac{r^{2}}{2}\arcsin\frac{y}{r}),$$

where *r* (= 0.6 mm) is the effective radius of the focused light spot, since the dimension of the QPD’s active area per element is 1.3 mm, and *p* is the light intensity that the QPD receives per unit area. The value of *p* is related to the total optical power from the LD, area of the light spot, and reflectivity and transmissivity of each optical component. The value of *p* for the angle sensor used in this research is 2.08 mW mm^−2^.

**Figure 3:**
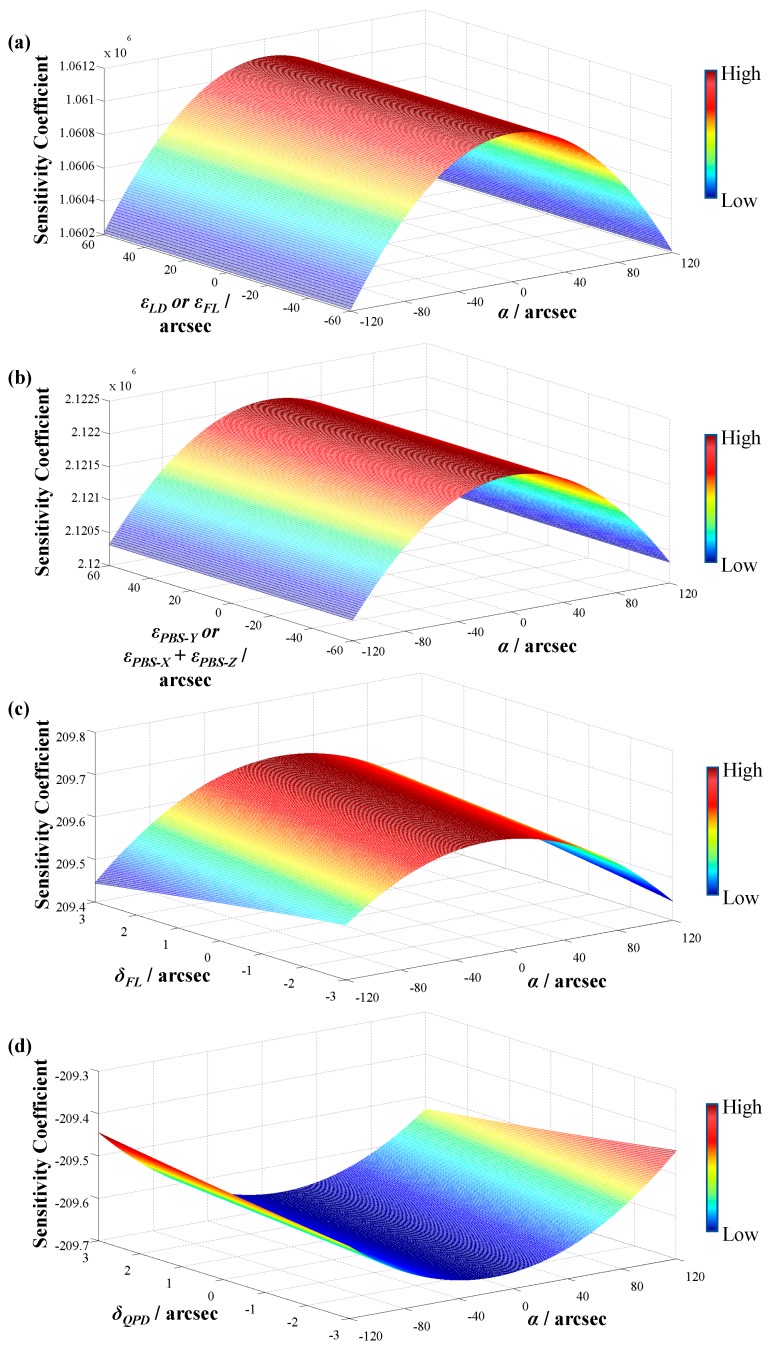
Sensitivity coefficients between the output voltage and (**a**) angular error of a focusing lens (FL) or laser diode (LD), (**b**) angular error of the polarizing beam splitter (PBS), (**c**) displacement error of FL, and (**d**) displacement error of the quadrant photodetector (QPD).

Substituting Equation (13) and Equation (14) and their relative parameters into Equation (17) and Equation (18), the sensitivities between each component’s angular error or displacement and the output voltage can be obtained by calculating the partial derivatives. The focal length of FL is 21 mm, and the measurement range of the angle sensor within which *α_Y_* and *α_Z_* change is ± 120 arcsec. Therefore, the sensitivity coefficients of each component to the output voltage can be obtained after considering just one error factor and giving others a value of zero, as shown in Figure 3. The rotation angles of PBS around the X and Z axes are considered together, since they only cause the focused light spot to move horizontally.

From Figure 3, it can be seen that: (1) the sensitivity coefficient of the PBS is twice that of the FL or the LD, and is 10^4^ times that of QPD; (2) the drift caused by the PBS is the largest, and the drift caused by the QPD can be negligible. Therefore, the fixed mounts of the PBS, FL, and LD should be considered to improve the sensor’s stability effectively.

### 3.2. Optimized Design and Simulation for Key Optical Mounts

The mounts’ deformations caused by temperature fluctuation and vibration will cause large displacement and angular errors for the fixed optical components. Therefore, in order to achieve a higher stability angle sensor, it is necessary to decrease the component mounts’ structural deformations. Some factors need to be considered before optimizing the design. Firstly, the yaw and pitch deformations of the optical components’ mounts should be reduced as much as possible for an angle sensor. Secondly, the symmetrical center point of each optical component can always be in one line, even if the temperature changes. Finally, the optical components should be placed and fixed on their mounts in a secure and stable way.

**Figure 4:**
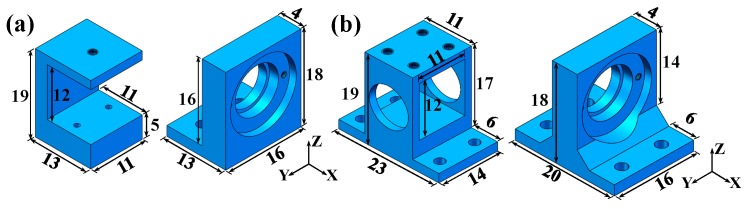
Fixed mounts of PBS and FL in (**a**) the original sensor and (**b**) optimized sensor.

Symmetrical design is often regarded as an effective method to evenly stress the structure and reduce the deflection of the devices. From Figure 3, we can see that the sensitivity coefficients of the PBS, LD, and FL cannot be ignored. Therefore, the fixed mounts of the PBS and FL were optimized in accordance with the symmetry structure. The distance for each mount from its lower surface to the lower surface of the fixed optical component is equivalent, so that the symmetrical center point of each optical component is always in one line. Other structural parameters of the mounts were obtained through many simulation analysis results. All optical elements were screwed onto the mounts using M1.6 screws. The original and optimized structures and sizes are shown in Figure 4. The original mount for the LD did not change, as it was already symmetrical.

**Figure 5:**
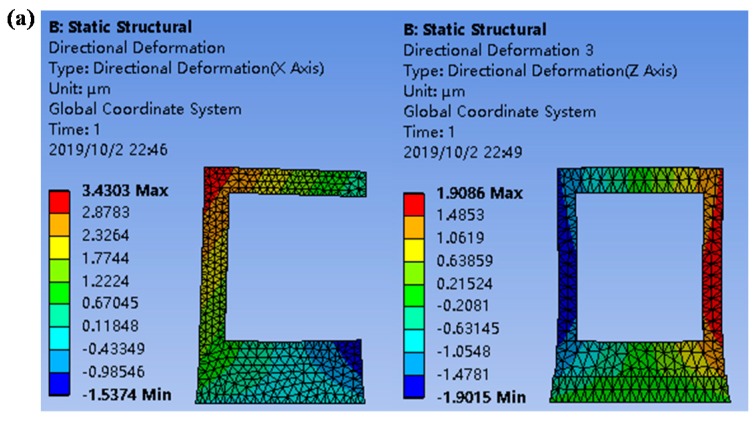

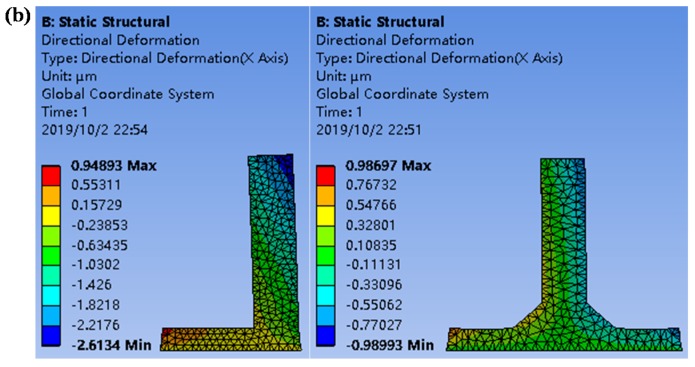
Thermal stability analysis results of the (**a**) original (left) and optimized (right) PBS mounts, and the (**b**) original (left) and optimized (right) FL mounts on the Z axis.

The mounts were analyzed by ANSYS 15.0 software, a large-scale general finite element analysis (FEA) software developed by American ANSYS company, to investigate their thermal stability characteristics. Considering that the working temperature of a sensor usually varies within a large range in practice, different temperatures ranging from ± 2 °C to ± 20 °C were selected for analysis, so as to make the results more convincing. Figure 5 shows the simulation results in the X direction when the temperature changed from 15 °C to 25 °C, from which the maximum deformations of the mounts could be obtained. Then, the angular errors could be estimated by dividing the maximum deformations by the effective lengths of the mounts in the Z direction. Table 2 shows the deformations and angular errors.

From Table 2, we can see that the angular error caused by the PBS in the XOZ plane decreased from 11.4 arcsec to 2.9 arcsec and that of the FL decreased from 6.1 arcsec to 1.3 arcsec when the temperature varied by 4 °C. The stability of both the PBS and FL was improved by more than 74% using the symmetrical design concept, even if the temperature varied by 40 °C.

The harmonic response analysis of the mounts was also analyzed using ANSYS 15.0 software. A vibration with an acceleration of 1900 mm s^−2^ and a frequency of 10 Hz was applied. All of the deformations of the mounts caused by the vibration were less than 4 nm, and so were neglected.

## 4. Experiments and Results

### 4.1. Calibration

**Figure 6:**
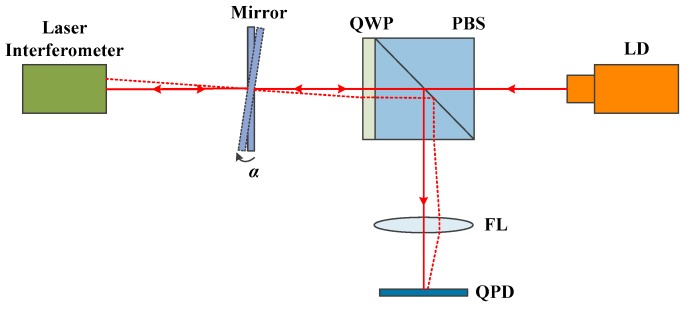
Schematic of the calibration setup.

**Figure 7:**
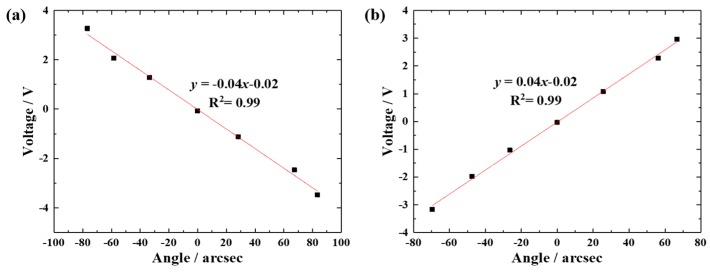
Calibrating results in the (**a**) X axis and (**b**) Y axis.

The 2D angle sensor was calibrated by the method shown in Figure 6. A laser interferometer (SP2000-TR, SIOS Meßtechnik GmbH, Ilmenau, German) was used as a reference. The calibration results are shown in Figure 7. From Figure 7, we can find that the sensor’s sensitivities are − 0.04 V/arcsec and 0.04 V/arcsec in the X and Y axes, respectively.

### 4.2. Thermal Stability Experiments

**Figure 8:**
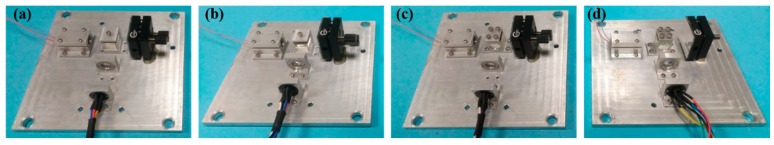
Photos of the (**a**) original sensor, (**b**) optimized sensor excluding the PBS mount, (**c**) optimized sensor excluding the FL mount, and (**d**) optimized angle sensor.

To verify the correctness of the error model and analysis results, comparative experiments for thermal stability were performed on the original sensor, optimized sensor excluding the PBS mount, optimized sensor excluding the FL mount, and optimized angle sensor. Experimental photos are shown in Figure 8.

**Figure 9:**
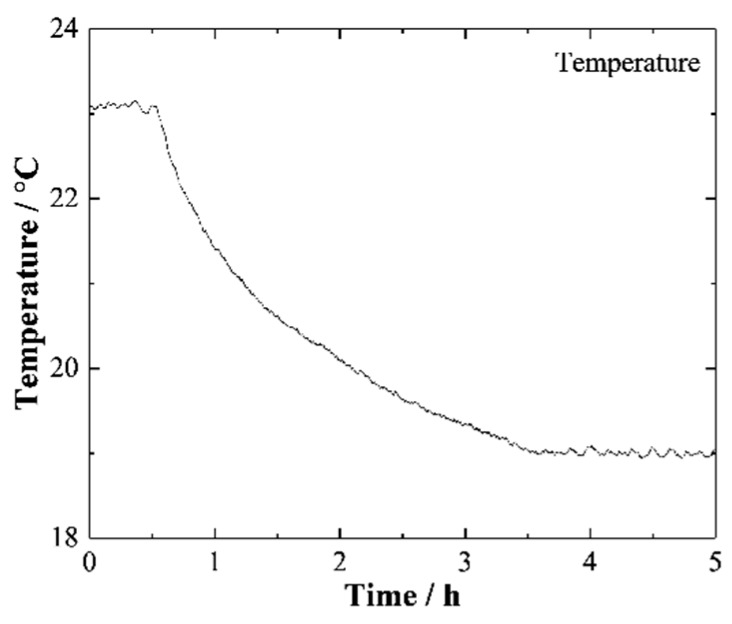
Temperature in the chamber.

**Figure 10:**
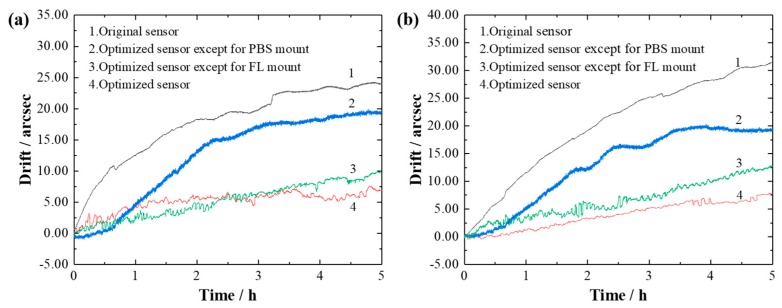
The drift of the sensors for (**a**) yaw and (**b**) pitch.

The four angle sensors were placed in a high-precision, constant-temperature chamber [13]. The steady-state temperature error of the chamber can be controlled within the range of ± 0.03 °C. Considering that the thermal deformation of a certain mount is linear with the temperature variation range, the temperature varied by 4 °C in all experiments conducted in this study. The initial temperature was set at 23.09 °C and then gradually decreased to 19.00 °C. The temperature in the chamber over five hours is shown in Figure 9. With the temperature decreasing, the drifts of the measured angles of each sensor were recorded, and are shown in Figure 10.

Similar trends are shown in Figure 10a,b. In comparison with the drifts of the original sensor (24.21 arcsec and 32.12 arcsec), the drifts of the optimized sensor were reduced to 7.66 arcsec and 8.36 arcsec. The thermal stability of the optimized sensor was improved by 68% to 74%. Under the same conditions, the drifts caused by the original PBS mount were 13.24 arcsec and 12.22 arcsec, while the drifts caused by the original FL mount were 3.36 arcsec and 5.27 arcsec. The former mount is more sensitive than the latter, and its drifts are more than twice those of the latter. The experimental results show that the stability of the angle sensor can be improved by at least 68% by using the symmetrical design concept, which is consistent with the previous mathematical model and simulation results. The difference could be caused by the errors in manufacturing, assembly, and experimentation.

### 4.3. Vibration Stability Experiments

**Figure 11:**
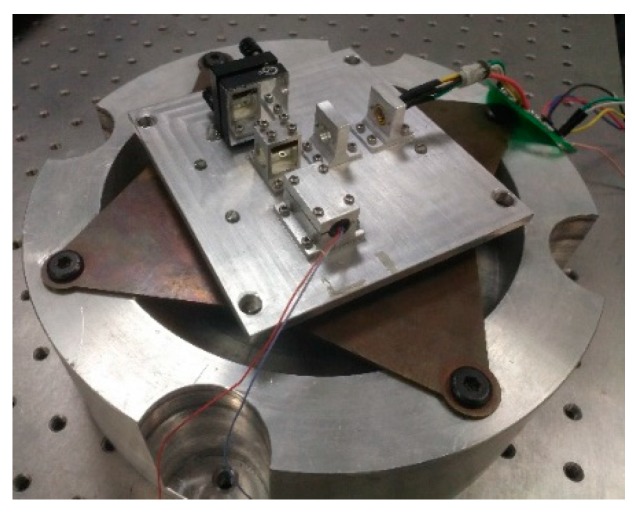
Picture of the vibration generator and angle sensor.

The angle sensors were placed on a low-frequency vibration generator (shown in Figure 11). Vibrations were generated by this generator [20], which was driven by a high-precision waveform generator and a power amplifier. Vibration stability experiments were carried out with sine waves.

**Figure 12:**
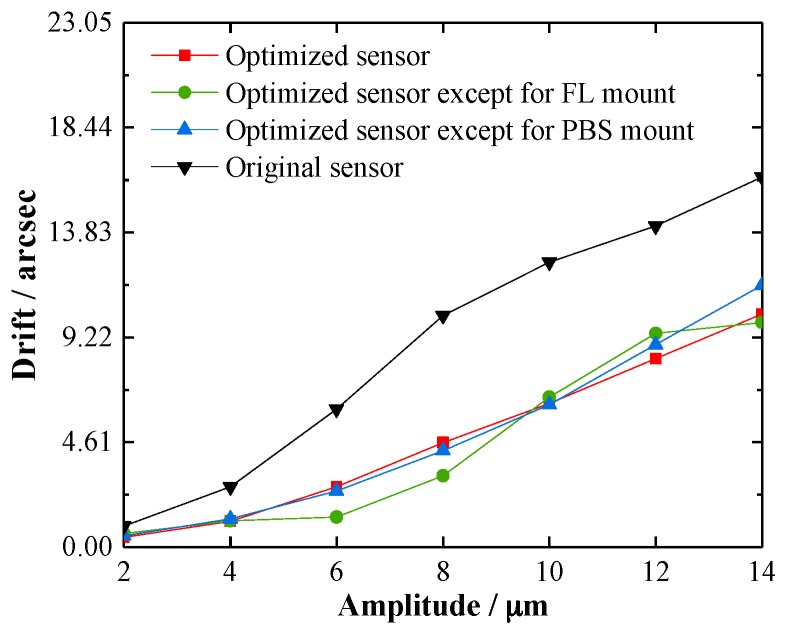
Drifts when a vertical vibration was applied.

Figure 12 shows the maximum drift errors when the generator applied a sine signal with a frequency of 10 Hz in a vibration amplitude range from 2 μm to 14 μm, since most vibrations can be reduced to this range by common isolation platforms. When the vibration amplitude was larger than 14 μm, the drift of the original sensor became almost constant because of the fabrication error, assembly clearance, and because the size of the structure has an upper limit. As seen in Figure 12, the angular drifts of both the original and optimized sensors became larger as the vibration amplitude increased. The drifts of the optimized sensors climbed more slowly than that of the original sensor, which illustrates that the wholly and partially optimized sensors are more stable when encountering a micro-vibration.

### 4.4. Drift Error Analysis

The thermal and vibration drifts of the sensor can be found in Figure 10 and Figure 12, respectively, and are listed in Table 3. Since the vibration drift experiment was not implemented for yaw, only the drift errors for pitch are evaluated here. Considering that thermal drifts and vibration drifts are generally independent, the synthesis drift errors were calculated using Equation (19):

(19) $$\delta_{s}=\sqrt{\delta_{t}^{2}+\delta_{v}^{2}},$$

where $\delta_{s}$ is the synthesis drift error of a sensor, $\delta_{t}$ is the maximum thermal drift error, and $\delta_{v}$ is the maximum vibration drift error.

From Table 3, it can be seen that the stability of the wholly optimized sensor was improved approximately 3 times. Moreover, the synthesis drift error of the optimized sensor excluding the FL mount was nearly equal to that of the wholly optimized sensor, which was smaller than for the original sensor and for the optimized sensor excluding the PBS mount. This means that PBS was the most sensitive component of the angle sensor and the optimization of the PBS mount was very effective in improving the stability. The results are completely consistent with the previous theoretical model and simulation results.

## 5. Conclusions

This paper presents a method to improve the stability of an angle sensor by combining mathematical analysis with symmetrical structures. Through mathematical analysis and simulation, error sensitivities were obtained for each element and key optical mounts requiring optimization were found. The symmetrical design method was used on these mounts to improve the stability of the sensor. The experimental results show that the structural deformation of the optical component mount can be reduced using the optimized design method. The sensor’s thermal stability was improved by approximately 3 times, and the sensor’s vibration stability was also improved by 35%. The experimental results also indicate that the effect of reflection devices on angle is greater than that of transmission devices. The proposed method is effective, low-cost, easy to carry out, and universally applicable. Future research will focus on how to further improve the measurement accuracy of such systems.

## Figures and Tables

**Table 1:** Comparison of previous methods for reducing drift.

Method	Drift	Advantage	Disadvantage	Reference
Constant temperature chamber	5 nm in 3 h	High accuracy	Bulky, high cost, high energy consumption	Li et al. [4,13]
Single-mode fiber-coupled lasers	Standard deviation: 0.86 μm in 0.5 h (X), 0.27 μm in 0.5 h (Y)	The laser’s drift is controlled	Other components’ drifts unconsidered	Feng et al. [14]
	± 0.3 arcsec in 55 min (pitch), ± 0.4 arcsec in 55 min (yaw)			Kuang et al. [15]
	Standard deviation: 6.035 μm in 3 h (X), 4.285 μm in 3 h (Y)			Hao et al. [16]
Angle Detection with single-cell photodiodes (SPDs)	—	High sensitivity and resolution	Stability unknown	Shimizu et al. [17]
Compensation with a common-path sensor	0.02 arcsec in 2 h	Passive compensation method	Complex, high cost	Zhu et al. [6]
Compensation with two piezoelectric actuators (PZTs)	± 0.01 arcsec	Real time compensation	Complex, high cost	Huang et al. [7]
Symmetrical structure	0.12 arcsec (temperature changes 5 °C)	Effective, convenient, and economic	Only thermal drift, wholly optimized with experience, suitable for simple sensors	Li et al. [18]

**Table 2:** The angular errors around the Y axis of the fixed mounts.

Temperature (20 °C)	±2	±5	±10	±20	—	±2	±5	±10	±20
Fixed Mount	Maximum Deformations (μm)				Effective Lengths (mm)	Angular Errors (arcsec)			
Original PBS mount	0.66	1.66	3.31	6.62	12	11.4	28.5	56.9	113.9
Optimized PBS mount	0.17	0.42	0.85	1.69	12	2.9	7.3	14.6	29.1
Original FL mount	0.47	1.19	2.37	4.75	16	6.1	15.3	30.6	61.2
Optimized FL mount	0.09	0.22	0.44	0.88	14	1.3	3.2	6.5	12.9

**Table 3:** Maximum drift errors of the angle sensors for pitch.

Type of the Angle	Maximum Drift Errors		
Sensors	$\delta_{t}$ (arcsec)	$\delta_{v}$ (arcsec)	$\delta_{s}$ (arcsec)
Original sensor	32.12	16.26	36.00
Optimized sensor except for PBS mount	19.90	11.50	22.98
Optimized sensor except for FL mount	13.63	9.87	16.83
Optimized sensor	8.36	10.24	13.22
